# Positive allosteric modulation of TRPV1 as a novel analgesic mechanism

**DOI:** 10.1186/1744-8069-8-70

**Published:** 2012-09-21

**Authors:** Evan E Lebovitz, Jason M Keller, Hal Kominsky, Krisztian Kaszas, Dragan Maric, Michael J Iadarola

**Affiliations:** 1 Neurobiology and Pain Therapeutics Section, Laboratory Of Sensory Biology, NIDCR, NIH, Bldg 49 Rm 1C2049 Convent Dr, Bethesda, MD, 20892, USA; 2 Laboratory of Neurophysiology, NINDS, NIH, Bethesda, MD, 20892, USA

**Keywords:** TRPV1, Pain, Capsaicin, Vanilloid, Nociception, Resiniferatoxin, ATF3, Dorsal root ganglion, MRS1477, Adelta fiber

## Abstract

**Background:**

The prevalence of long-term opiate use in treating chronic non-cancer pain is increasing, and prescription opioid abuse and dependence are a major public health concern. To explore alternatives to opioid-based analgesia, the present study investigates a novel allosteric pharmacological approach operating through the cation channel TRPV1. This channel is highly expressed in subpopulations of primary afferent unmyelinated C- and lightly-myelinated Aδ-fibers that detect low and high rates of noxious heating, respectively, and it is also activated by vanilloid agonists and low pH. Sufficient doses of exogenous vanilloid agonists, such as capsaicin or resiniferatoxin, can inactivate/deactivate primary afferent endings due to calcium overload, and we hypothesized that positive allosteric modulation of agonist-activated TRPV1 could produce a selective, temporary inactivation of nociceptive nerve terminals *in vivo*. We previously identified MRS1477, a 1,4-dihydropyridine that potentiates vanilloid and pH activation of TRPV1 *in vitro,* but displays no detectable intrinsic agonist activity of its own. To study the *in vivo* effects of MRS1477, we injected the hind paws of rats with a non-deactivating dose of capsaicin, MRS1477, or the combination. An infrared diode laser was used to stimulate TRPV1-expressing nerve terminals and the latency and intensity of paw withdrawal responses were recorded. qRT-PCR and immunohistochemistry were performed on dorsal root ganglia to examine changes in gene expression and the cellular specificity of such changes following treatment.

**Results:**

Withdrawal responses of the capsaicin-only or MRS1477-only treated paws were not significantly different from the untreated, contralateral paws. However, rats treated with the combination of capsaicin and MRS1477 exhibited increased withdrawal latency and decreased response intensity consistent with agonist potentiation and inactivation or lesion of TRPV1-containing nerve terminals. The loss of nerve endings was manifested by an increase in levels of axotomy markers assessed by qRT-PCR and colocalization of ATF3 in TRPV1^+^ cells visualized via immunohistochemistry.

**Conclusions:**

The present observations suggest a novel, non-narcotic, selective, long-lasting TRPV1-based approach for analgesia that may be effective in acute, persistent, or chronic pain disorders.

## Background

During the past decade, the use of opiates for chronic pain management has expanded from mainly postoperative and oncological patients to include those with chronic non-cancer pain. Consequently, the abuse of and dependence on prescription opioids has become an increasing public health concern [1-5]. Given the detrimental side effects and addiction potential of these medications, it is critical that we develop novel, non-opiate based pharmacological agents to treat chronic pain. One potential therapeutic avenue involves targeting TRPV1, a sodium/calcium ion channel highly expressed in a subpopulation of unmyelinated C- and lightly-myelinated Aδ-afferent primary afferent neurons [6-9]. This receptor responds to multifactorial inputs and is activated by temperature >43°C [7], protons [7], exogenous vanilloid ligands such as capsaicin and resiniferatoxin (RTX) [7], endogenous vanilloids such as NADA [10], the endocannibinoid anandamide [11,12], and various fatty acids [13-15]. It is also sensitized by post-translational modifications through the actions of algesic molecules produced after tissue injury or during inflammation [16-20], and the plasma membrane expression of TRPV1 is greatly upregulated in small- to medium-diameter sensory neurons during inflammation [21,22]. Extensive drug development has been directed at generating antagonists of the TRPV1 orthosteric capsaicin-binding site for acute and chronic pain. This research yielded many outstanding pharmacological agents in several chemical classes [23] that include, for example, ABT-102 [24], SB-705498 [25], AS1928370 [26], and AMG8562 [27]. However, as a class, this group of antagonists encountered difficulties in transitioning to clinical utilization due to two main side effects. The first is an unpredictable level of hyperthermia; the propensity for which is variable among individual agents [28]. The second is that TRPV1 antagonists potently block hot temperature sensation throughout the body and the loss of cutaneous sensation to noxious thermal stimuli can place these patients at risk for accidental burn injuries [28].

In an attempt to circumvent some of the issues with current TRPV1 antagonists, novel reversible or permanent interventional neuroablative therapies based on TRPV1 agonists are being explored [29,30]. Administration of a vanilloid agonist, such as capsaicin or its ultrapotent analog RTX [31], can cause calcium-induced cytotoxicity and lead to a TRPV1-selective axonopathy that spares surrounding non-TRPV1-expressing somatosensory proprioceptive afferent and motor efferent nerve fibers. The potency and selectivity of vanilloid agonists for TRPV1 afferents has been demonstrated repeatedly *in vivo*[8,32-40]. For example, ablation of TRPV1^+^ nerve terminals occurring after a single subcutaneous injection of RTX into the rat hind paw results in prolonged but reversible analgesia that can be detected for one to several weeks and induces up-regulation of molecular markers for axon damage/repair in neuronal perikarya of the dorsal root ganglia [8,32]. We have also found that topical application of RTX onto the cornea results in temporary loss of the capsaicin eyewipe response in parallel with a loss of CGRP immunoreactive afferents in stromal fiber bundles [41]. While the actions of peripherally administered RTX are localized and reversible, RTX given intrathecally produces a spatially broader effect over multiple dermatomes with permanent pain relief [33,34,36]. The therapeutic efficacy of intrathecal RTX is especially evident in veterinary canine patients with naturally-occurring osteosarcoma [34], and RTX delivered intrathecally to treat advanced cancer pain in humans is currently in a Phase I clinical trial (<http://clinicalstudies.info.nih.gov/detail/A_2009-D-0039.html>). At present, the only available FDA-approved agonist-based treatments contain capsaicin in an over-the-counter low dose (0.075%) topical cream and a high dose (8%) prescription cutaneous patch [42]. Topical therapy is reported to be effective for post-herpetic neuralgia [35], HIV-associated distal sensory polyneuropathy [43], and painful diabetic neuropathy [44]. Because TRPV1 agonists are painful initially upon application, in general, administration of a TRPV1 agonist necessitates pretreatment with a local anesthetic and, in the case of intrathecal administration, general anesthesia, which requires the patient to be either in the clinic or in the operating room. In addition, while capsaicin is effective when administered focally to treat Morton’s neuroma or osteoarthritis [45] and RTX is effective when administered intrathecally to treat osteosarcoma [34], they generally cannot be given systemically in large doses since they produce a decrease in core body temperature and may be cardiotoxic, although there is a much larger safety margin for RTX [29]. Thus while some conditions may be amenable to local, interventional TRPV1 agonist administration, other pain conditions, where pain is more diffuse or delocalized, may not be appropriate candidates for treatment with localized therapies.

**Figure 1:**
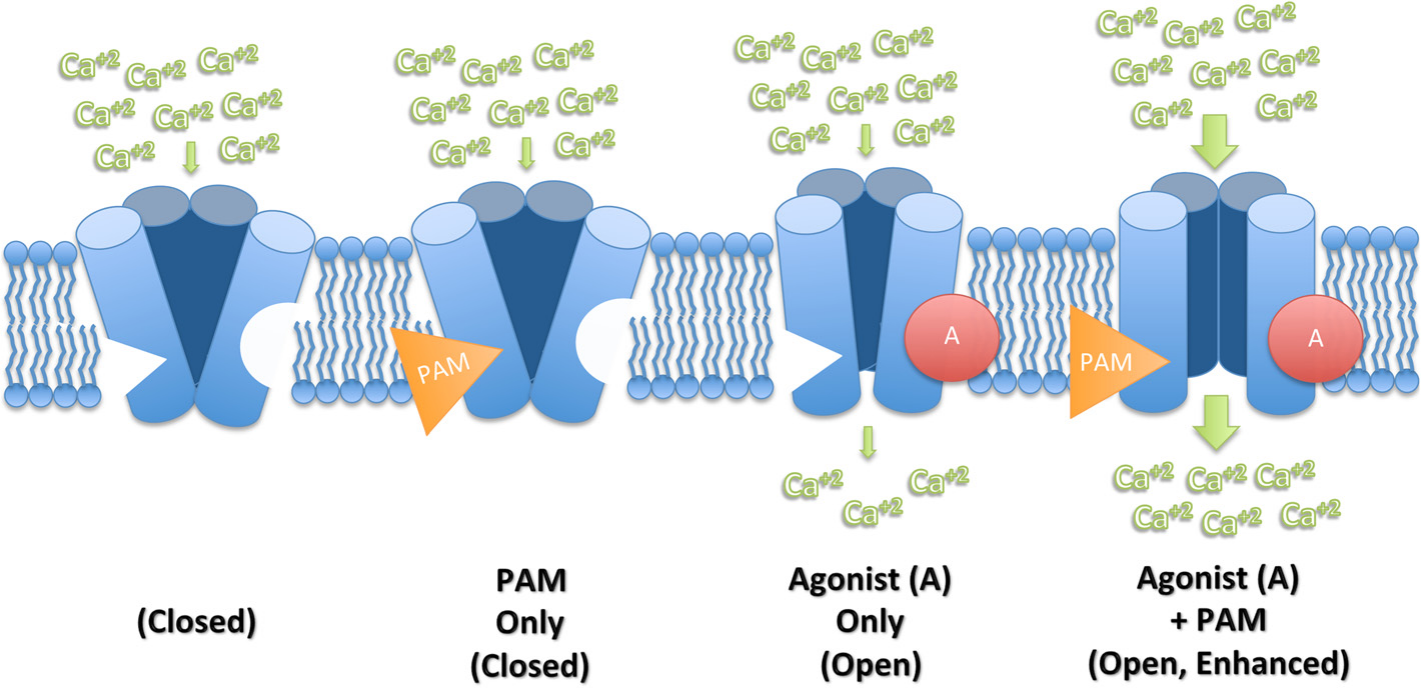
**Conceptualized model of PAM modulation directly on TRPV1.** Homotetrameric TRPV1 is permeable to cations, notably sodium and calcium, and possesses an orthosteric binding site for capsaicin and an allosteric binding site. At baseline, no agonist is bound and the channel is closed (far left). Binding of a positive allosteric modulator does not activate channel opening (mid left). When an agonist such as capsaicin binds (mid right), a conformational change allows sodium and calcium to flow into the neuron resulting in depolarization and, if threshold is reached, generation of an action potential. When capsaicin binds, the allosteric site targeted by a PAM (far right) then becomes functionally accessible. The PAM increases cation influx and the likelihood of axonal depolarization. Sufficient depolarization can result in calcium-induced cytotoxicity leading to inactivation of the conductive potential of the axon and analgesia.

TRPV1 is highly conductive, can integrate various noxious stimuli, and is located in a clinically relevant population of nociresponsive afferent neurons; these characteristics make it an important therapeutic target. In an effort to combine the ease of systemic administration of antagonists with the potent, long-lasting analgesic qualities of agonists, we are exploring positive allosteric modulation of TRPV1 as a novel analgesic mechanism [46]. An ideal TRPV1 positive allosteric modulator (PAM) would have no intrinsic activity but would enhance receptor activation by an orthosteric agonist and, as a result, would act conditionally to potentiate only TRPV1 on afferent nerves terminating in regions of tissue damage or inflammation where channels are highly active (see Figure 1). By over-driving the channel, a PAM acting directly on TRPV1 could induce calcium-mediated toxicity in the active nerve terminals or axons leading to functional inactivation or ablation and ultimately to a focused, selective, long-lasting analgesia. Here, we present *in vivo* evidence that it is possible to positively modulate agonist-activated TRPV1-expressing nerve terminals to the point of deafferentation. MRS1477, a small molecule 1,4-dihydropyridine that we have previously identified [47] and characterized [46]*in vitro*, was used to potentiate capsaicin-activated nerve terminals in the rat hind paw. We examined analgesic effects of terminal inactivation by measuring acute thermal nociception and changes in gene expression markers of peripheral nerve terminal ablation, damage, or axotomy in lumbar dorsal root ganglia. Our data demonstrate a rapid, spatially localized ablation of TRPV1-expressing nerve terminals after a single, subcutaneous injection resulting in sustained analgesia lasting several days. The present observations suggest a novel, non-narcotic, selective, long-lasting TRPV1-based approach for pain reduction that may be effective in acute, persistent, or chronic pain disorders.

## Methods

### Animals

Male Sprague–Dawley rats (250–400g) were housed under a 12h light–dark cycle and allowed access to food and water *ad libitum*. The ambient temperature of the holding and testing rooms was ~22°C. Procedures were performed in accordance with the National Institutes of Health (NIH) Guidelines for the Care and Use of Laboratory Animals, and approved by the National Institute of Dental and Craniofacial Research (NIDCR) Animal Care and Use Committee. All efforts were made to minimize both animal numbers and distress within the experiments.

### Drug solutions and administration

The drug vehicle for all experiments consisted of 7.5% Tween-80 (Sigma-Aldrich, P8074) and 0.05% ascorbic acid (Spectrum Chemical, AS105) in PBS, at pH 7.2. Resiniferatoxin from stock (“RTX”, custom purification, 100 μg/ml in vehicle) was further diluted in vehicle to 200 ng/100 μl for injection. A 20 mM stock solution of MRS1477 (FW = 389.51, [47]) was prepared in 100% DMSO (Sigma-Aldrich, D2650) and further diluted in vehicle to either 1 μg/100 μl (25 μM) or 2 μg/100 μl (50 μM), where specified. Capsaicin (“CAP”, Sigma-Aldrich, M2028) was prepared as a 100 mM stock solution in absolute ethanol, stored at −80°C, and was diluted directly into vehicle on the day of the experiments to 30 μg/100 μl. CAP-only injectates contained an equal amount of DMSO as those with MRS1477. All subcutaneous injections were made using a 29G×1/2", 3/10 cc insulin syringe (Terumo Medical Corp., Cat No. SS*30 M2913). The experimenter was blinded to the identity of the injectates in the various behavioral experiments.

### Behavioral measurements

Behavioral assessments were performed as reported previously [8]. Briefly, unrestrained rats were placed on a clear glass platform under a small plastic cage (23×13×13 cm), which allowed them to move freely. Groups of rats were habituated to the testing apparatus for a minimum of three days by placing them under the cages on the glass platform and given both Aδ- and C-fiber laser stimuli. On the day of testing, rats were allowed to habituate for at least 10 min prior to thermal stimulation. Rats were tested prior to injection to establish a baseline, then at 2, 24, and 48 h post-injection. An infrared diode laser (Lasmed LLC, Mountain View, CA) was aimed at the ventral hind paw, with a perpendicular approach to the plantar skin. Aδ-fibers were preferentially stimulated with a short-pulse (100 msec) using a high-intensity small-diameter (1.6 mm) beam, and behavioral responses were assessed on a five-point scale: 0 = no response, 1 = orient to stimulus, 2 = orient with paw lift, 3 = lift paw, orient, and shake, and 4 = lift paw with lick [8]. Only responses graded as 2 or above included a paw withdrawal. Aδ-fibers were stimulated with laser intensities ranging from 3.59 W/mm^2^ to 5.61 W/mm^2^. C-fibers were preferentially stimulated with a continuous, wide-diameter (5 mm), low-energy pulse (0.083 W/mm^2^) and paw withdrawal latency (sec) served as the primary endpoint. One cohort of rats (n = 4) receiving 2 μg MRS1477 + 30 μg CAP was tested repeatedly for 24 days to evaluate the time course for recovery of thermal sensitivity.

### qRT-PCR analysis

Rats were euthanized 24 h post-injection. The L4, L5, and L6 dorsal root ganglia were dissected and pooled as either ipsilateral or contralateral to the drug injection and then frozen on dry ice. Total RNA was extracted using Qiazol (Qiagen, Cat No. 79306) and the RNeasy Kit (Qiagen, Cat No. 74104) with a DNase I digestion step, all according to the manufacturer’s instructions. RNA integrity was verified using an Agilent 2100 Bioanalyzer. All total RNA samples had an RNA integrity number (RIN) between 8.3 and 9.5 with an average of 8.9. cDNA was synthesized from total RNA using the High Capacity cDNA Reverse Transcription Kit (Applied Biosystems, Cat No. 4368813) and Random Primers. Quantitative Real-Time PCR (qRT-PCR) was performed using Power SYBR Green Mastermix (Applied Biosystems, Cat No. 4309155) on a Stratagene Mx3000P. Additionally, Reverse Transcription PCR (RT-PCR) was performed using the Access RT-PCR system (Promega, Cat No. A1250). RT-PCR products were resolved on a 2% agarose gel with ethidium bromide staining. All oligonucleotide primer sequences are listed below (Table 1). The results were normalized to GAPDH. Comparisons of gene expression from ipsilateral vs. contralateral DRG were made using a paired Student’s *t*-test. Markers of axotomy included activating transcription factor 3 (“ATF3”; [48]), neuropeptide Y (“NPY”; [49]), galanin (“GAL”; [50]), vasoactive intestinal polypeptide (“VIP”; [51-53]), and monocyte chemoattractant protein 1 (“MCP-1”; [54]).

**Table 1:** RT-PCR primer pairs and PCR product sizes

**Gene**	**Primer pairs**	**Product (bp)**
NPY	CCGCCCGCCATGATGCTAGG	150
	GGCCATGTCCTCTGCTGGCG	
GAL	CCTCGTGCGCTTCCCTACGC	148
	GCCCCTGGCCATCTTGAGCA	
ATF3	GGACGACCGACCAACCCGC	122
	CATTTTGCTCCAGTCTTCGCTCGGG	
VIP	GGACCAGGGGCAGACTCCGT	121
	TCCATCTCGGTGCCTCCTTGGG	
MCP-1	GAGGCCAGCCCAGAAACCAGC	153
	TGGGGCATTAACTGCATCTGGC	
GAPDH	GGGGCTCTCTGCTCCTCCCTG	108
	ACGGCCAAATCCGTTCACACC	

### Statistical analyses

Comparisons of gene expression from ipsilateral and contralateral tissues were made by paired Student’s *t*-test. For other single comparisons between two different groups, unpaired t-tests were used. For multiple comparisons, one-way and two-way ANOVA tests were used with, respectively, Tukey’s post-hoc test and Bonferroni multiple comparisons. For ordinal response score data from behavioral ratings that were used to evaluate Aδ-fiber responses, the Mann–Whitney U test was used. Differences were considered significant where p < 0.05. Time versus drug effects for longitudinal behavioral data were analyzed using two-way ANOVA.

### Immunohistochemistry

Rats used for immunohistochemistry were deeply anesthetized with sodium pentobarbital and transcardially perfused with cold PBS followed by 4% paraformaldehyde. The L4-L5-L6 dorsal root ganglia were harvested bilaterally, post-fixed in 4% paraformaldehyde for 2 h, cryopreserved in 20% sucrose, then mounted and frozen for sectioning on a cryostat.

All DRG tissues were sectioned at 10 μm and mounted directly onto slides and stored at −80°C until use. Prior to staining with antibodies, the sections were thawed at RT and allowed to air dry. The entire immunostaining procedure was carried out at RT. Sections were washed in HEPES Buffer (“HB”; see below) and fixed in 4% paraformaldehyde for 10 min, then washed in HB and counterfixed in absolute methanol for 5 min. Antigen retrieval was performed by incubation of sections in 1% SDS for 5 min and the tissues were then incubated in Background Buster solution (Innovex Biosciences, Cat No. NB306-50) for 20 min to block non-specific antibody binding. Sections were subsequently washed in HB and then incubated with primary antibodies of interest (see below) diluted in HB for 1 h in a humidified chamber. The unbound antibodies were washed away using HB and the sections were then incubated with appropriate fluorescently-labeled secondary antibodies (see below) for 1 h in a dark, humidified chamber. Finally, the sections were washed in HB to remove unbound antibodies, sealed with Immuno Mount (GeneTex, Cat No. GTX30928) and a coverslip, and were viewed the following day.

All sections were imaged using a fluorescence microscope (Axiovert 200 M; Carl Zeiss, Thornwood, NY) equipped with a 20× Plan-Apochromat objective (Carl Zeiss), a high-resolution cooled digital camera (ORCA-ER; Hamamatsu Photonics, Japan), a 100 W mercury-arc lamp light source and wavelengths selected with excitation/dichroic/emission filter sets (Semrock, Rochester, NY) optimized to detect the following fluorophores: DAPI, Alexa Fluor 488, and Alexa Fluor 546. Each labeling reaction was captured using appropriate fluorescence filter sets and the images individually digitized at 12-bit resolution using the Volocity image acquisition program (Improvision Inc., Lexington, MA). An appropriate color table was applied to each image to either match its emission spectrum or to set a distinguishing color balance. The pseudocolored images were then converted into TIFF files, exported to Adobe Photoshop, and overlaid as individual layers to display multi-colored merged composites.

### HEPES Buffer (HB)

HB was made fresh prior to staining and kept at 4°C. Buffer was made in 1 liter batches and contained 145 mM NaCl, 5 mM KCl, 0.8 mM MgCl_2_, 1.8 mM CaCl_2_, 10 mM HEPES, and 0.1% BSA. The solution was filter sterilized and the pH was adjusted to 7.3 with NaOH/HCl.

### Antibodies

Polyclonal primary antibodies consisted of anti-TRPV1 (guinea pig, 1:100, Novus Biologicals, Cat No. NB300-122) and anti-ATF3 (rabbit, 1:200, Santa Cruz Biotechnology, Cat No. sc-188). Both primary antibodies were diluted with glycerol 1:1 prior to use to limit degradation following repeated freeze-thaw cycles. Fluorescently labeled secondary antibodies were purchased from Invitrogen and consisted of goat anti-guinea pig Alexa Fluor 488 (1:200, Cat No. A-11073) and goat anti-rabbit Alexa Fluor 546 (1:200, Cat No. A-11035). All antibodies were diluted from stock solutions to working concentrations with HB.

## Results

In order to evaluate the *in vivo* effects of MRS1477, we measured nociceptive responses to thermal stimuli in Aδ- and C-fibers after mid-plantar injections into rat hind paws. The CAP dose was determined empirically in pilot studies. To do this, we sought a dose that produced robust nocifensive responses from the rats upon acute injection (vocalization, paw shaking, paw licking, and erythema), but did not cause subsequent analgesia when tested with noxious thermal stimuli the next day. We found 30 μg of CAP in 100 μl to be effective for this purpose. The MRS1477 doses were based on our previous *in vitro* data, and we were primarily interested in finding a minimum effective dose when administered with 30 μg of CAP. Response scores (Aδ stimulus, 3.59-5.61 W/mm^2^) and paw withdrawal latencies (C-fiber stimulus, 0.083 W/mm^2^) were evaluated prior to and at 2, 24, and 48 h following subcutaneous injection of either MRS1477 alone, CAP alone, or the co-administration of MRS1477 and CAP. We chose the starting point of 2 h to minimize acute effects of the injection on response sensitivity and also to allow time for denervation to occur [32]. A separate cohort of rats (n = 6) was used to compare MRS1477 alone to untreated contralateral paws, and the behavioral data were not found to be significantly different (not shown). Throughout the rest of the experiments, CAP alone and untreated contralateral paw data served as controls.

**Figure 2:**
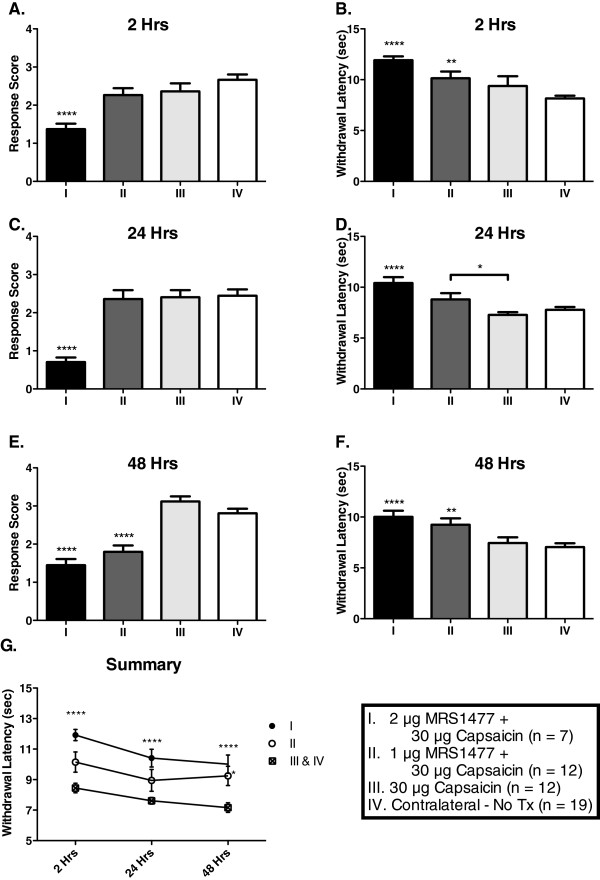
**Prolonged attenuation of responses to noxious thermal stimuli following intraplantar co-administration of MRS1477 and capsaicin.** An infrared diode laser was used to preferentially stimulate Aδ- (**A**,**C**,**E**) or C-fibers (**B**,**D**,**F**,**G**) in the hind paw following intraplantar drug administration. At 2 h post-injection (**A**,**B**), rats treated with 2 μg MRS1477 + 30 μg CAP showed decreased nociceptive responses to both Aδ- and C-fiber stimuli, while rats treated with 1 μg MRS1477 + 30 μg CAP showed a significant attenuation of only C-fiber responses when compared to untreated controls. At 24 h post-injection (**C**,**D**), rats treated with 2 μg MRS1477 + 30 μg CAP still showed decreased responses to both Aδ- and C-fiber stimuli, and again 1 μg MRS1477 + 30 μg CAP-treated rats showed a significant increase in C-fiber withdrawal latency only. At 48 h post-treatment (**E**,**F**), rats receiving either 1 or 2 μg MRS1477 + 30 μg CAP displayed significantly reduced nociceptive responses under both stimulus paradigms when compared to 30 μg CAP-treated and untreated controls. Longitudinal C-fiber data are displayed for comparison (**G**). Since they were not significantly different from each other, data from the untreated and 30 μg CAP-only cohorts were combined in (**G**). * = p < 0.5; ** = p < 0.01; *** = p < 0.001; **** = p < 0.0001.

Attenuation of response sensitivity to acute thermal stimulation was apparent as early as 2 h post-injection (Figure 2A). The mean Aδ response score for 2 μg MRS1477 + 30 μg CAP (1.4 ± 0.1, n = 7) was significantly lower (p < 0.0001) than 30 μg CAP alone (2.4 ± 0.2, n = 12), or the untreated contralateral paw (2.7 ± 0.1, n = 19). At 2 h and a dose of 1 μg MRS1477 + 30 μg CAP, the Aδ response score (2.3 ± 0.2, n = 12) was not significantly different than untreated contralateral paw. The mean C-fiber evoked paw withdrawal latency (Figure 2B) after 2 μg MRS1477 + CAP (11.9 ± 0.4 s, n = 7) was significantly elevated (p < 0.0001) compared to CAP alone (9.4 ± 1.0 s, n = 12), or the untreated contralateral paw (8.2 ± 0.3 s, n = 19). Withdrawal latencies after 1 μg MRS1477 + CAP (10.2 ± 0.7 s, n = 12) were significantly higher (p < 0.01) than untreated paws (8.2 ± 0.3 s, n = 19). Importantly, neither capsaicin alone nor MRS1477 alone produced significant changes in response sensitivity.

Paws treated with MRS1477 + CAP showed a loss of thermal sensitivity that persisted at 24 h (Figure 2C). The mean Aδ response score after 2 μg MRS1477 + 30 μg CAP (0.7 ± 0.1, n = 7) was significantly lower (p < 0.0001) than all other treatments; the mean response score was 2.4 ± 0.2 (n = 12) after 1 μg MRS1477 + 30 μg CAP and 2.4 ± 0.2 (n = 12) after CAP alone, which was not significantly different from the untreated contralateral paws (2.4 ± 0.2, n = 19). The mean C-fiber evoked paw withdrawal latency at 24 h (Figure 2D) after 2 μg MRS1477 + CAP (10.4 ± 0.6 s, n = 7) were significantly greater (p < 0.0001) than CAP alone (7.3 ± 0.3 s, n = 12), and compared to contralateral paws (7.8 ± 0.3 s, n = 19). The mean latency for 1 μg MRS1477 + CAP was significantly higher (p < 0.05) than CAP alone. At this time, the increase in latency produced by 2 μg MRS1477 + 30 μg CAP was not significantly different than that seen with 1 μg MRS1477 + 30 μg CAP (8.8 ± 0.6 s, n = 12).

Blunted nocifensive behavioral responses continued for 48 h and were now apparent with tests of Aδ fibers at both doses of MRS1477 (Figure 2E). The mean Aδ response scores after 1 μg MRS1477 + CAP (1.8 ± 0.2, n = 12) and 2 μg MRS1477 + CAP (1.5 ± 0.2, n = 7) were similar, but they were both significantly lower compared to CAP alone (3.2 ± 0.1, n = 12) and untreated contralateral paws (2.8 ± 0.1, n = 19) (p < 0.0001). At 48 h (Figure 2F), the mean C-fiber withdrawal latencies after 1 μg MRS1477 + CAP (9.2 ± 0.6 s, n = 12) and 2 μg MRS1477 + CAP (10.0 ± 0.6 s, n = 7) were both significantly greater (p < 0.01 and p < 0.0001, respectively) than CAP alone (7.4 ± 0.6 s, n = 12), and the untreated contralateral paws (7.0 ± 0.4 s, n = 19). A summary of the dose–response effect at 2, 24, and 48 h for the C-fiber stimuli is shown in Figure 2G. We combined CAP alone with the untreated contralateral paw data at each time point, since the CAP only injection had no effect. Again, the loss of acute thermal sensitivity after 2 μg MRS1477 + CAP is significant at all time points (p < 0.0001), while loss of thermal sensitivity in the 1 μg MRS1477 + CAP treatment group was significant at the 48 h test (p < 0.05). The persistent nature of the analgesia provided the initial indication that TRPV1-expressing afferent nerve endings are being ablated by the combined treatment.

**Figure 3:**
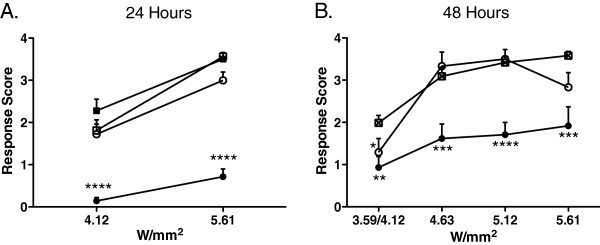
**Paw withdrawal response as a function of Aδ stimulus intensity.** Rat hind paws were stimulated at 24 and 48 h following intraplantar drug treatment. After 24 h (**A**), 2 μg MRS1477 + 30 μg CAP-treated hind paws showed significant analgesia to stimuli at both power settings compared to 30 μg CAP-only and untreated paws (p < 0.0001). After 48 h (**B**), 2 μg MRS1477 + 30 μg CAP-treated hind paws displayed significant analgesia at all power settings compared to 30 μg CAP-only and untreated controls. Since they were not significantly different from each other, data from the untreated contralateral and 30 μg CAP-only paws were combined in (B). ●2 μg MRS1477 + 30 μg CAP (n = 7). ○1 μg MRS1477 + 30 μg CAP (n = 12). ■30 μg CAP (n = 12). □Contralateral - No Tx (n = 19). ⊠30 μg CAP & Contra (n = 14).

In order to test the degree of efficacy of the PAM treatment, we examined Aδ-evoked responses over a range of increasing laser intensities at 24 h (Figure 3A) and 48 h (Figure 3B) post-injection. At 24 h, Aδ response scores were significantly attenuated at both 4.12 W/mm^2^ and 5.61 W/mm^2^ after treatment with 2 μg MRS1477 + CAP compared to either 1 μg MRS1477 + CAP or the controls (Figure 3A; Mann–Whitney U test, p < 0.0001). At 48 h, Aδ response scores were recorded at stimulus intensities ranging from 3.59–5.61 W/mm^2^. Rats receiving 2 μg MRS1477 + CAP continued to show significantly attenuated response scores at all intensities compared to controls, while rats receiving 1 μg MRS1477 + CAP showed attenuated response scores at the lowest laser intensities (Figure 3B; Mann–Whitney U test).

**Figure 4:**
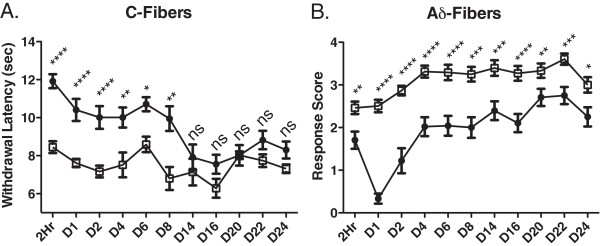
**Longitudinal recovery of acute thermal pain sensation following intraplantar MRS1477 + capsaicin.** Rats (n = 4) were injected with 2 μg MRS1477 + 30 μg CAP unilaterally and were followed behaviorally for 24 days. Contralateral paws served as the untreated controls. Sensitivity to C-fiber stimulation (**A**) returned more quickly (by day 14) than did sensitivity to Aδ stimulation (**B**). * = p < 0.05; ** = p < 0.01; *** = p < 0.001, **** = p < 0.0001. ●2 μg MRS1477 + 30 μg CAP (n = 4). □Contralateral - No Tx (n = 4).

One group of rats (n = 4) was followed longitudinally after unilateral injection of 2 μg MRS1477 + 30 μg CAP (Figure 4). Reduced sensitivity to both C- and Aδ-fiber stimulation was apparent by 2 h. This attenuation peaked between 1 and 2 days under both stimulus paradigms, remained significantly different for at least 8 days using the C-fiber stimulus (Figure 4A, two-way ANOVA), but with the Aδ stimulus, sensitivity was significantly reduced for approximately 24 days (Figure 4B, Mann–Whitney U test). This is consistent with observations from our previous study with RTX [8] that TRPV1-expressing Aδ-fibers and nociceptive behaviors associated with their activation may be more susceptible to long term inactivation compared to C-fibers. The combination of PAM and agonist produces a long-lasting effect similar to that of RTX, a potent vanilloid agonist.

**Figure 5:**
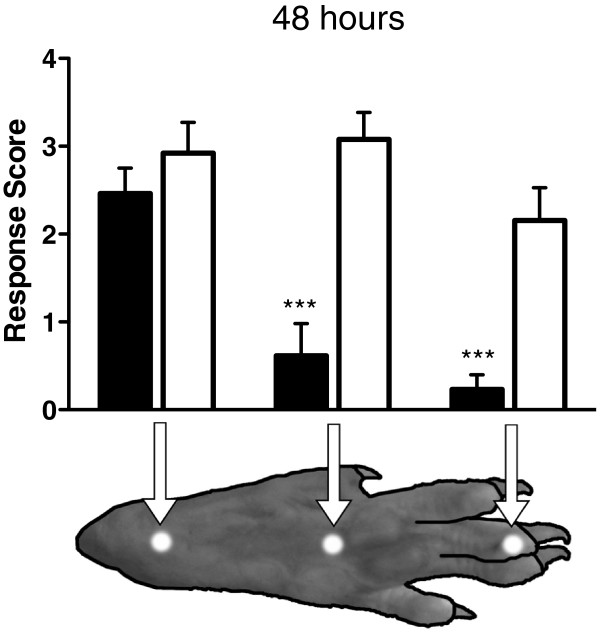
**Pronounced attenuation of thermal sensitivity in mid-plantar and distal locations after mid-plantar injection of MRS1477 + capsaicin.** Thermal sensitivity was mapped across the plantar surface of rat hind paws with the small-diameter Aδ laser stimulus. Withdrawal response scores were determined by subjective scoring on a 0 to 4 scale (see Methods). Nocifensive responses were significantly attenuated by treatment with 2 μg MRS1477 + 30 μg CAP as compared to untreated paws in the mid-plantar region and distally in the toes, but not in the heel. Testing was performed 48 h after injection using a laser stimulus intensity of 4.12 W/mm^2^. The beam diameter (1.6 mm) is shown to scale. ■2 μg MRS1477 + 30 μg CAP (n = 3). □Contralateral - No Tx (n = 3).

The small diameter beam of the Aδ stimulus allowed detailed spatial mapping of thermal sensitivity across the plantar surface of the hind paw. All rats treated with 2 μg MRS1477 + 30 μg CAP were given a mid-plantar injection in 100 μL total volume, and this resulted in a pronounced loss of sensitivity in the toes (p < 0.001) and mid-plantar (p < 0.001) area of the foot pad, but there was no detectable effect at the heel (Figure 5, Mann–Whitney U test). These results demonstrate that inactivation occurred not only at the injection site but also on axons running through the injection site projecting distally into the toes. These data are consistent with observations recorded after intraplantar RTX, and suggest that axonal effects are a common feature of both PAM and vanilloid agonist actions [8].

**Figure 6:**
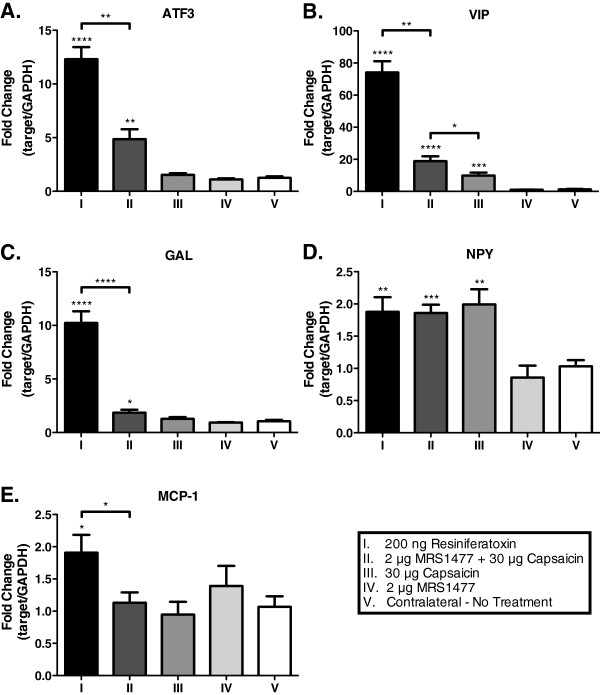
**Gene markers for axotomy are upregulated in lumbar dorsal root ganglia after intraplantar MRS1477 + capsaicin.** Rats received a hind paw injection of either 200 ng RTX, 30 μg CAP, 2 μg MRS1477, or 2 μg MRS1477 + 30 μg CAP and were compared to untreated paws. After 24 h, whole lumbar DRG (L4-L6) were removed, RNA was isolated, and expression of ATF3 (**A**), VIP (**B**), GAL (**C**), NPY (**D**), and MCP-1 (**E**) was quantified via qRT-PCR. Data were normalized to the reference gene GAPDH and then represented as the fold change relative to the untreated, contralateral DRG. Injection of MRS1477 + CAP or RTX caused a significant upregulation of ATF3, VIP, GAL, and NPY expression compared to either CAP-only, MRS1477-only, or untreated cohorts. Expression levels of VIP and NPY were also significantly upregulated following CAP-only treatment. MCP-1 expression was upregulated after RTX treatment, but not after MRS1477 + CAP.

We have previously observed that the potent neurotoxin RTX, due to the resultant nerve terminal axonopathy induced in TRPV1-expressing afferent nerve endings, produces an up-regulation of genes associated with nerve damage and/or repair in the ipsilateral dorsal root ganglion [8]. Therefore, we hypothesized that if MRS1477 + CAP acted similarly in producing local nerve terminal ablation, our behavioral data would correlate with increased expression of the same molecular markers in the DRG. Rats (n = 3 per group) were injected unilaterally with 200 ng RTX, 30 μg CAP, 2 μg MRS1477, or 2 μg MRS1477 + 30 μg CAP. The contralateral paw was not treated and DRG from this side served as controls. After 24 h, the rats were euthanized and the L4, L5, and L6 DRG were isolated and pooled for each group. After extraction of total RNA, qRT-PCR was performed to assess changes in transcript levels for several markers (Figure 6). Expression of ATF3 (Figure 6A) was greatly increased after treatment with either RTX (p < 0.0001) or 2 μg MRS1477 + CAP (p < 0.01), while no significant changes were seen after either CAP alone or MRS1477 alone when compared to the controls. VIP expression (Figure 6B) was greatly upregulated by RTX (p < 0.0001), and less so but significantly by 2 μg MRS1477 + CAP (p < 0.0001), and to a lesser extent by CAP alone (p < 0.001). Although CAP induced VIP expression, the MRS1477 + CAP group was significantly higher than CAP alone (p < 0.05). Both RTX (p < 0.0001) and MRS1477 + CAP (p < 0.05) produced a significant increase in GAL expression compared to controls (Figure 6C), while neither CAP alone nor MRS1477 alone produced upregulation. NPY expression was also induced by RTX, CAP alone (Figure 6D; p < 0.01), and by MRS1477 + CAP (p < 0.001). RTX treatment upregulated MCP-1 (Figure 6E; p < 0.05), but CAP alone, MRS1477 alone, and MRS1477 + CAP were not different from controls.

**Figure 7:**
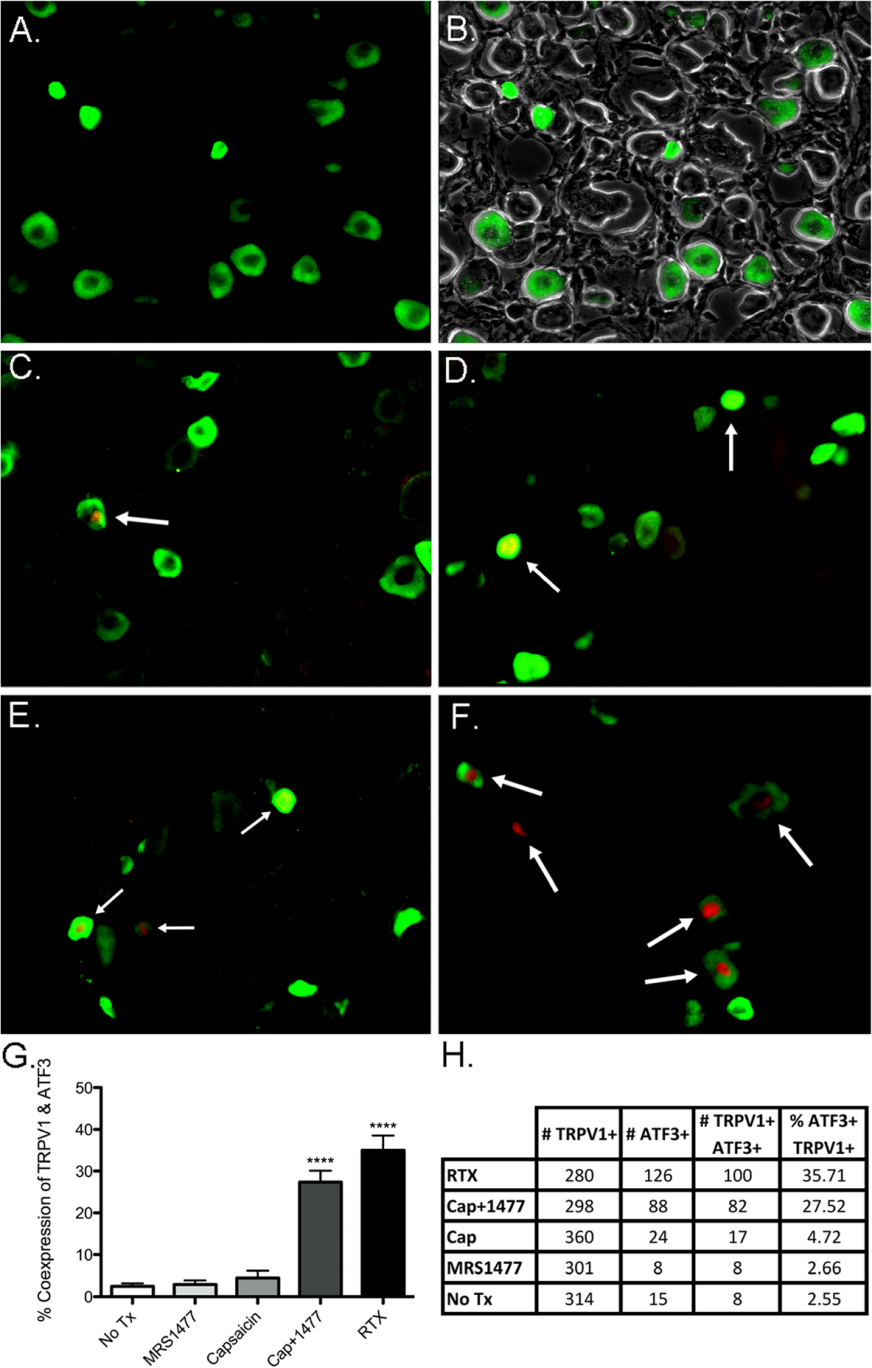
**Lumbar dorsal root ganglia neurons co-label with TRPV1 and ATF3 after intraplantar MRS1477 + capsaicin.** Double-immunolabeling of TRPV1 (green) and ATF3 (red) is shown in rat DRG neurons from fixed-frozen sections (**A-F**). Rats were either not treated (**A**, **B** with phase), or given an intraplantar injection of 2 μg MRS1477 (**C**), 30 μg CAP (**D**), 2 μg MRS1477 + 30 μg CAP (**E**), or 200 ng RTX (**F**). ATF3 appears as red fluorescence concentrated in neuronal nuclei. Yellow nuclei result from an overlap of ATF3^+^ and TRPV1^+^ staining. ATF3^+^ nuclei were manually counted and calculated as the ratio of ATF3^+^/TRPV1^+^ cells over the total number of TRPV1^+^ cells in a given field (**G**). All treatment groups were compared statistically to the untreated controls using an unpaired Student’s *t*-test (**** = p < 0.0001). Results of cell counts from 10 fields analyzed at 200× magnification are summarized (**H**). Arrows indicate representative co-labeled cells.

To determine if the effect of MRS1477 on peripheral nerve terminals was specific to TRPV1^+^ neurons, we double-labeled DRG neurons via immunofluorescence for TRPV1 and ATF3 (Figure 7). A group of rats (n = 3) was injected with 200 ng RTX into one hind paw while the contralateral side was left untreated. An additional group of rats (n = 3) was injected with 30 μg CAP into one hind paw and 2 μg MRS1477 + 30 μg CAP into the contralateral paw. One additional group of rats (n = 3) was injected with 2 μg MRS1477 alone. After euthanasia at 24 h, dorsal root ganglia were removed and immunostained as described in the Methods. The TRPV1 antibody stained a sub-population of small- to medium-diameter DRG cell soma (Figure 7A,B). Approximately 2 to 3 fields of TRPV1^+^ neurons were digitally captured from a single ganglion at 200× magnification and used for counting cells. ATF3 is not expressed in normal sensory neurons, but after axotomy, ATF3 expression is greatly upregulated in damaged neurons [48]. In the untreated controls, ATF3 staining was diffuse or non-apparent (Figure 7A,B). We found positive ATF3 staining in the nuclei of TRPV1^+^ neurons from each treatment group (Figure 7H), but both the MRS1477 + CAP (Figure 7E) and the RTX (Figure 7F) groups showed much greater TRPV1/ATF3 co-labeling compared to CAP (Figure 7D), MRS1477 (Figure 7C), or untreated contralateral paws (Figure 7A, p < 0.001 and p < 0.0001, respectively; Figure 7G). In animals treated with RTX, approximately 35% of the TRPV1^+^ neurons contained ATF3^+^ nuclei, and in the CAP + MRS1477 group, 27% of the neurons contained ATF3^+^ nuclei. This contrasts with ~3-5% of TRPV1^+^ neurons that colocalized ATF3 in the no treatment, MRS1477 alone, and CAP alone groups.

## Discussion

The present data demonstrate the ability of MRS1477, a positive allosteric modulator of TRPV1, to potentiate the effect of capsaicin (CAP) *in vivo* resulting in a rapid peripheral nerve terminal inactivation and a significant, long-lasting yet reversible analgesia to cutaneous noxious thermal stimulation in rats. Neither MRS1477 nor capsaicin alone produced suppression of behavioral responses. Evidence for loss of the cutaneous nerve terminals was reinforced by parallel molecular studies of gene expression changes in DRG. The corresponding cellular specificity of the effect was examined by immunohistochemical double labeling for ATF3 (a nuclear stain) and TRPV1 (largely cytoplasmic). Intraplantar injection of the combination of MRS1477 and CAP produced colocalization of TRPV1 and ATF3 in neurons in lumbar DRG. These data indicate that potentiation of agonist-activated TRPV1 by a positive allosteric modulator can produce a localized nerve terminal inactivation *in vivo* that yields rapid and prolonged analgesia.

We previously demonstrated that MRS1477 exhibits no agonist activity at TRPV1, but potentiates the calcium influx induced by orthosteric vanilloid agonists and TRPV1 stimulation by protons or sensitization by phorbol ester [46,47]. In the previous study, we also observed MRS1477 potentiation of intraperitoneal capsaicin-induced hypothermia [46]. In the present study, we were able to induce analgesia through functional inactivation of peripheral primary afferent nerve terminals similar to the long-lasting effect of the potent TRPV1 agonist RTX, which served as our positive control [8]. Inhibition of the response to noxious thermal stimuli was observed after administration of 2 μg MRS1477 plus 30 μg capsaicin, while 1 μg MRS1477 plus 30 μg capsaicin produced a less robust response, consistent with a dose-dependent relationship. Furthermore, the effect of the agonist-PAM combination was spatially discrete. When administered as a mid-plantar injection, the resulting analgesia occurred at the treated area and distally in the toes with relative sparing of the heel (see Figure 5). This localized analgesia in the mid-plantar and distal regions suggests that the nerve terminal inactivation is confined to the nerve endings in the immediate vicinity of the injection site and to axons projecting through it; an effect we have demonstrated previously with peripherally administered resiniferatoxin [8]. We refer to the process involving nerve endings as “nerve terminal inactivation” or “deactivation,” rather than complete ablation, since the nerve endings can grow back and become active again [32,55]. We also distinguish inactivation/deactivation from desensitization, the latter referring to the progressive decrement in response upon multiple, successive applications of a vanilloid agonist; desensitization is an endpoint frequently investigated in electrophysiological experiments. This inactivation process can form a new potential approach to analgesic pharmacology that is conditional and pain-state dependent [30].

When we tracked the recovery of Aδ-mediated thermal nociception, we observed substantial yet incomplete recovery by day 4, followed by a gradual, progressive return towards baseline over the next 20 days. With C-fiber stimulation, there was significant attenuation of nociceptive responses over the first 6 to 8 days and a convergence with control latency measurements by day 14. This slow return to baseline is consistent with an earlier study of the effects of RTX on cutaneous Aδ- and C-fiber TRPV1-expressing nerve terminals [8]. These data support the notion that a TRPV1 PAM, in combination with an agonist in a non-deactivating dose, can produce a long-lasting analgesia that shares many of the behavioral and afferent fiber-type specific characteristics obtained by treatment with the potent, selective agonist RTX.

Gene upregulation of markers for neuronal damage in dorsal root ganglia also provides evidence that PAM treatment modifies the integrity of primary afferent nerve terminals. This was explored using two approaches: transcript amplification by qRT-PCR and immunohistochemical staining for relevant proteins and neuropeptides. Increased transcript levels of nerve damage biomarkers were measured in the dorsal root ganglia for the transcription factor ATF3 [48] and the neuropeptides vasoactive intestinal peptide (VIP) [51-53], galanin (GAL) [50], and neuropeptide Y (NPY) [49]. We previously demonstrated that these molecules can also be induced by injection of RTX into the hind paw; a result consistent with chemo-axotomy of the TRPV1-expressing nerve endings. By extension, we hypothesized that the PAM/agonist combination would produce a similar effect. Our qRT-PCR data demonstrated that MRS1477 plus CAP resulted in an induction of ATF3, VIP, NPY, and GAL transcripts. In contrast, neither CAP nor MRS1477 alone induced upregulation of any of the biomarkers except NPY and VIP, which were upregulated after CAP alone. This may be related to the low but detectable basal expression levels of NPY compared to the other genes, which are nearly undetectable at basal state. For VIP, while induction by CAP is higher than in the untreated contralateral DRG, treatment with MRS1477 + CAP produces both significantly greater VIP induction and behavioral effects compared to CAP alone.

We determined that the molecular axotomy was specific to TRPV1^+^ neurons by immunofluorescence double labeling using antibodies targeting TRPV1 and ATF3. Both the behavioral and molecular data suggest that MRS1477 is working on TRPV1-expressing afferent terminals. We reasoned that this could be further substantiated if the elevation in ATF3 was enriched in TRPV1-expressing neurons in the DRG. Figure 7 shows that in rats treated with MRS1477 and CAP, nuclear staining for ATF3 is found predominantly in TRPV1 expressing neurons: 93% (82/88) of ATF3 positive nuclei co-localized to TRPV1-stained neurons, and this percentage is similar following positive control injections of RTX (79%, 100/126). However not all TRPV1^+^ neurons expressed ATF3. Likely, these represent TRPV1^+^ neurons with axons terminating in the lower limb but not in the treated plantar surface of the hind paw. Extensive co-localization of axotomy-related molecular alterations to TRPV1^+^ neurons suggests a high degree of cellular specificity for MRS1477. At the same time, the rats did not exhibit autotomy behaviors that commonly accompany sciatic nerve transection [56-58]. These results are consistent with earlier studies on peripheral injections of RTX into the hind paw [32], perineurally [38,55], or directly into rat or monkey trigeminal [33,59]. These data suggest that our TRPV1 PAM is producing the expected on-target effects and does not produce unexpected sensory abnormalities.

Evidence from this experiment and our previous RTX studies [8,32] suggest that TRPV1-expressing Aδ-fibers are more susceptible to long-term denervation. The greater structural complexity of lightly myelinated Aδ-fibers could account for the apparent disparity in functional recovery time compared to unmyelinated C-fibers [60]. Also, the paranodal location of the plasma-membrane calcium ATPase [61], a major regulator of intracellular calcium levels in myelinated DRG neurons [62,63], may accentuate calcium toxicity in Aδ-fibers. It is interesting to speculate whether differential efficacy at the two fiber types could form a useful strategy for treating pathological pain problems.

Allosteric modulation is an important approach to consider in therapeutic drug development [64]. Currently, allosteric modulation of GABA_A_ ligand-gated ion channels is a well-exploited mechanism for general anesthetics, benzodiazepine pre-anesthetic medications, and anxiolytics [65-67]. Agonist potentiation is also being explored to mitigate the side effects of poorly tolerated but otherwise effective analgesics acting on, for example, GABA_B_ receptors [68] and nicotinic acetylcholine receptors [69]. Conversely, negative allosteric modulation of glutamate receptors has been explored clinically for the treatment of migraine [70]. Our studies investigate MRS1477 as a proof-of-concept molecule for allosteric potentiation of TRPV1. We have demonstrated that MRS1477 can effectively modulate CAP activation *in vitro* and *in vivo*, and while CAP is not a physiologically relevant agonist, our model serves as a framework for future testing of animal pain models. In pathological states there are several variables that can influence PAM efficacy. TRPV1 PAM actions are predicated on the existence and generation of endogenous TRPV1 activators (endovanilloids) that are adequately potent and present in sufficient concentrations at sites of tissue damage or inflammation to bind to the orthosteric vanilloid site; low pH also plays an activating role [71,72]. A model for the interactions between TRPV1, PAMs, and endovanilloids is presented in Figure 1, which shows a diagram of four potential states of the receptor: closed, both sites unoccupied; closed with PAM site occupied; open with vanilloid site occupied; and open with both sites occupied. It may be that only certain pain conditions generate sufficient endovanilloids or protons to be permissive for positive allosteric modulation of TRPV1. In chronic pain states, nociceptive nerve endings remain active and do not transition into an inactive state without pharmacological intervention, since the endings have multiple mechanisms to cope with transmembrane calcium flux [62]. Thus, despite a low pH and an endovanilloid-enriched environment, the added “push” from a PAM would be necessary to achieve nerve terminal inactivation [46].

Perhaps the greatest advantage of a TRPV1 PAM in terms of clinical pharmacology is the mechanism of calcium-mediated nerve terminal inactivation, which can potentially produce an effective, long-term analgesia. Even the most highly specific TRPV1 antagonist blocks just one ligand-gated ion channel, leaving numerous other TRPV1-independent receptors on the nerve endings available for stimulation by algesic mediators. In contrast, nerve terminal inactivation renders the fiber essentially insensitive to a variety of noxious stimuli. Thus, several practical and theoretical characteristics of a TRPV1 PAM offer the potential for a new, emerging class of analgesics.

## Competing interests

The authors declare that they have no competing interests.

## Authors’ contributions

EEL and JMK conducted experiments, analyzed data, and drafted the manuscript. HK conducted RNA extraction and PCR experiments. KK worked on manuscript and provided expertise on PAM pharmacology. DM provided immunocytochemical analysis. MJI coordinated study and worked on data analysis and the manuscript. All authors read and approved the final manuscript.

## Funding

EEL received funding from the Howard Hughes Medical Institute as an HHMI-NIH Research Scholar and from the Division of Intramural Research NIDCR, NIH. This work was supported by the Division of Intramural Research, NIDCR, NIH. NIH grant R03MH089480.
